# Hunting the Best Opportunity Through the Arrow of General Decision-Making Styles: Unfolding the Role of Social Capital and Entrepreneurial Intention

**DOI:** 10.3389/fpsyg.2022.814424

**Published:** 2022-03-03

**Authors:** Jiang Hong, Shabeeb Ahmad Gill, Hina Javaid, Qamar Ali, Majid Murad, Muhammad Shafique

**Affiliations:** ^1^Shenzhen Shining Sea Asset Management Co., Ltd., Shenzhen, China; ^2^Lyallpur Business School, Government College University, Faisalabad, Pakistan; ^3^Riphah International University, Islamabad, Pakistan; ^4^Government Fatima Jinnah Women Hospital, Multan, Pakistan; ^5^School of Management, Jiangsu University, Zhenjiang, China

**Keywords:** decision-making style, entrepreneurial intentions, discovery and creation, social capital, entrepreneurial opportunity, healthcare entrepreneurship, second-order moderated mediation model

## Abstract

This research aims to identify the investor’s decision-making styles and their impact on entrepreneurial opportunities through the mediation role of entrepreneurial intention and moderation effect of social capital in the healthcare sector of Pakistan. This study applied a structural equation modeling (SEM) to test the hypotheses on a sample of 400 healthcare investors. Our findings reveal that the conditional indirect relationship of entrepreneurial intention through social capital was significant on opportunity creation and an insignificant influence on opportunity discovery from decision-making styles. This study provides implications for policymakers to enhance entrepreneurial opportunity creation by providing robust social environment investors and encouraging them to create business ventures in the healthcare sector.

## Introduction

The twenty-first century human beings are not well equipped in healthcare; the COVID-19 taught us the proper lesson (Spoorthy et al., 2020). The situation of developed countries is not acceptable in healthcare. The pandemic has proved it. So, there is a big question mark on developing countries’ current and future conditions. However, healthcare facility is the intrinsic right of all the citizens and a key tool to reduce poverty (Javed et al., 2019). The healthcare entrepreneurial intention leads toward socioeconomic prosperity and increases the life expectancy ratio (Weiss et al., 2019). The developing countries can bring enormous economic hustle in their GDP through healthcare venture creation. Population wise, Pakistan is the fifth largest country, and its GDP rank is 45th in the World, which is alarming (Mohammad et al., 2007). One can better understand the healthcare conditions of the country’s two hundred and twenty million people with low per capita income, increasing inflation, unsecured food situation, and unemployment (Ruiz-Rosa et al., 2020). So, there is a drastic need to make a massive investment in the healthcare sector of Pakistan.

Moreover, Ayub et al. (2018) argue that the government of Pakistan took the incumbent step to control the country’s healthcare situation by introducing family health insurance in the province of Khyber-Pakhtunkhwa (KP), where each family has the healthcare insurance of Rs. 7,50,000 rupee. They can get this facility on their computerized national identity cards (CNICs) in every public and private sector hospital. The same facility has been announced for the biggest province of Punjab, with around one hundred and twenty million. In two districts, Prime Minister (PM), along with the Chief Minister (CM), has inaugurated this facility in May 2021 and promised to cover the entire province till December 2021 (Abdullah et al., 2021). It would be a landmark success in the healthcare sector of Pakistan and exemplary for all the developing countries. This project has enormous potential for private sector investors to come and join the party. Now, the point is how the entrepreneurs perceive this opportunity and unfold it.

There is an extensive debate between entrepreneurship researchers and practitioners about why some investors invest in entrepreneurial opportunities, and some do not because they are there (Rastkhiz et al., 2018; Reuber et al., 2018). Casson and Wadeson (2007) started a debate and discussed that the competitive imperfections start due to discovery theory after changes in the market environment. González et al. (2017) stated that there is zero correlation between the theory of discovery and individual differences in previous research to exploit an entrepreneurial opportunity (Worrall, 1985). Furthermore, it was understood that different entrepreneurial opportunity and discovery depends upon their attribute’s variations (Klein, 2008). It revealed that the decision to exploit opportunity is due to the relationship between individual differences and discovery theory (Li et al., 2020b). It became challenging to examine the individual differences and variations in opportunity simultaneously, so this approach becomes irrelevant to entrepreneurial general decision-making styles (GDMSs) (Jones and Barnir, 2019; Vial and Richomme-Huet, 2021).

The entrepreneurs make decisions in a precarious context because the discovery theory leads to opportunities associated with the innovative objectives (Martin and Wilson, 2016). On the other hand, the creation theory leads entrepreneurs to create opportunities through profound research and exploration, which is the only difference between entrepreneurial and non-entrepreneurial intention (Alvarez and Barney, 2007). Nevertheless, this is also a very ambiguous decision-making process for investors with entrepreneurial intentions (Burgers and Van de Vrande, 2016). Therefore, this study has taken two theories in entrepreneurial decision-making and opportunity for new venture creation. Prior researchers believe that opportunity discovery and creation theories of entrepreneurship are complementary to study combined to get more insight into new business development (Alvarez and Barney, 2007; Shu et al., 2018).

An ideal situation would be that theories of opportunity discovery and creation gave a clear picture of entrepreneurial decision-making related to the new business opportunities (Puhakka, 2007). Smith et al. (2019) argued that opportunity creation has different entrepreneurial decision-making and business planning assumptions. Opportunity discovery has various applications in decision-making related to the same phenomena (Laferriere et al., 2019). The unfolding role of social capital and entrepreneurial intention also makes this phenomenon more complex (Weiss et al., 2019). A concise and adoptable decision-making model would enhance the confidence level of investors to select a suitable opportunity strategy. The categorical position of social capital and entrepreneurial intention leads to attain the most appropriate investment opportunity (Henley et al., 2017). The investors need to cash the social capital to clear their entrepreneurial intention to start a new healthcare venture. This robust venture creation improves the investment in the country’s healthcare sector and creates employment opportunities.

The role of social capital as a regional factor could play a vital role in the entrepreneurial intention, and its moderating role could enhance investors’ intention in the new venture creation or entrepreneurial actions (Shu et al., 2018). Furthermore, this study contributes to entrepreneurship literature into the following perspectives; first, this research is the first empirical study to explore the best opportunity strategy between discovery and creation to fill the literature gap (Alvarez and Barney, 2007). Second, this study urges to establish the unfolding role of social capital and entrepreneurial intention through a second-order moderated mediation model. Third, this study contributes to the literature of opportunity theories, clarifies the role of opportunity discovery and opportunity creation concerning the healthcare sector, and discusses the importance of decision-making in selecting the best opportunity strategy. Fourth, this study provides a unique statistical approach to run a complex conceptual model in a single click that would enhance the credibility of the empirical investigation.

This study strived to address the potential research question.

RQ1: What is the influence of general decision-making styles GDMS on entrepreneurial intention, opportunity discovery, and creation?

The remainder of this article organizes as follows: first part discusses the literature review and hypotheses development. Subsequently, we exalt the research methodology through a research journey followed by empirical data analysis and result elaboration. Finally, we conclude this research by discussing the theoretical implications for researchers, academia, and practical implications for investors and policymakers.

## Literature Review and Hypotheses Development

Looking into previous studies, the role of opportunity discovery and opportunity creation is introduced as the potential, influential component of entrepreneurship toward new venture creation (Chetty et al., 2018; Shu et al., 2018). After that, the scholarly debate has shifted from entrepreneurial opportunities to identify social opportunities that extended the influence of social capital on entrepreneurial intention (Weiss et al., 2019). Li et al. (2020a) explored the entrepreneurial intention related to the theory of planned behavior, but the theories of discovery and creation less attention due to their ambiguous literary background. However, in the past, studies have confirmed that opportunity creation and opportunity discovery are mutually exclusive theories, and these theories are also different in their analysis of the origin (Alvarez and Barney, 2007; Martin and Wilson, 2016). Entrepreneurs’ decision-making styles could best define the actual entrepreneurial intention (Krasniqi et al., 2019).

### General Decision-Making Styles and Entrepreneurial Intention on Opportunity Discovery With Social Capital

The theory of discovery and creation has different ties with opportunities, environments, and resources (Davidsson et al., 2020). According to the discovery’s perspective, a prospect drives entrepreneurial action based on current market conditions (Alvarez and Barney, 2007). It considered that the opportunities are floating in the environment; the way to discover through changes in technology and consumer demands. The discovery of an opportunity is not long-lasting anyone in the future can discover the same opportunity because this is the age of connectivity and media (González et al., 2017).

Moreover, Li et al. (2020b) remarked that entrepreneurship is difficult to study without considering entrepreneurial intention because it is a better tool to measure new venture creation. Nevertheless, entrepreneurship-related practitioners and researchers are now under stress because they have understood that the scope of entrepreneurial intention is very narrow to start a new business in the future (Bogatyreva et al., 2019). Furthermore, Neumeyer et al. (2019) defined social capital in literature as the source of providing shared representation, interpretation, and system of meaning among entrepreneurs’ social networks. These components developed an entrepreneurial culture where high social legitimacy leaders have entrepreneurial minds (Kibler et al., 2014).

To make a decision, the authors studied GDMSs as a single component to understanding how to convert the information in decision-making rather than consider it a cognitive style (Curşeu and Schruijer, 2012; Krasniqi et al., 2019). The decision-making styles have been defined as a “*habitual pattern used by individuals when making decisions”* (Citroen, 2011, p. 11). The decision-making styles have been precisely elaborated by Scott and Bruce (1995), the learned, habitual response pattern exhibited by an individual when confronted with a decision situation. According to Krasniqi et al. (2019) GDMSs can potentially influence entrepreneurial opportunity, and investors must consider the decision-making styles for opportunity discovery and creation (Foss et al., 2015). Therefore, based on discussion, the following hypotheses are formulated;

H1a: There is a positive relationship between GDMSs and entrepreneurial intention.

H1b: There is a positive relationship between entrepreneurial intention and opportunity discovery.

H1c: There is a positive relationship between decision-making styles and opportunity discovery.

H1d: The influence of entrepreneurial intention on opportunity discovery will be moderated by social capital such that upper levels of social capital will enhance the positive effect of entrepreneurial intention on opportunity discovery.

### General Decision-Making Styles and Entrepreneurial Intention on Opportunity Creation With Social Capital

Alvarez and Barney (2007) argued that discovery theory has been well established in the literature, although the theory of creation has not yet to be articulated as a single coherent theory. Entrepreneurial intention leads to the new venture action; the theory of creation is a logical alternate of discovery based on the theoretical background (Klein, 2008; Pret and Carter, 2017). Moreover, Lins and Doktor (2014) discussed that opportunities could be created by investing in technology and the market environment for wealth creation, but it is expensive and not good enough for new business formation. Meanwhile, Park et al. (2017) illustrated that opportunity creation sometimes results in uncertain market situations and becomes a challenge to extract upon opportunity seekers’ experience and knowledge. Compared to the discovery theory, the opportunity creation theory believes that entrepreneurial opportunities do not exist unless created (Alvarez and Barney, 2007; Yu et al., 2021). In regional-level societies, due to complex hierarchies, it becomes difficult to share the available resources, but if there are strong ties, people tend to share their support for the sake of opportunity creation to start a new business venture (Chandra et al., 2009). As a result, the entrepreneurs became more selective in their social enactment process, making connections based on their experience and market analysis. As a result, they know when to utilize their social capital for opportunity creation and new venture action (Weiss et al., 2019).

**FIGURE 1:**
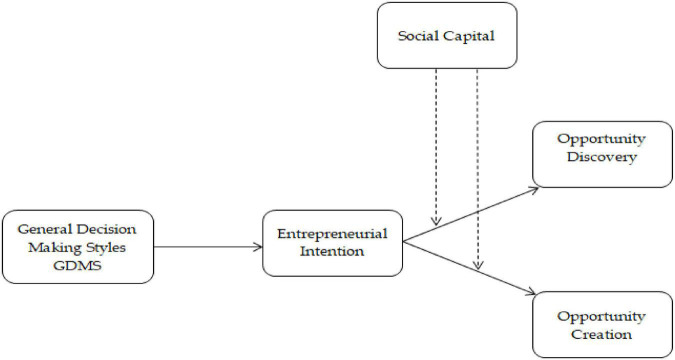
Conceptual model.

Krasniqi et al. (2019) used GDMSs to measure the respondents’ decision-making tendency, but very few of them connected the GDMS with entrepreneurial opportunities. The patterns of association between five styles theoretically measure entrepreneurs’ decision-making skills; it can be studied as a single component (Spicer and Sadler-Smith, 2005). The decision-making process is uncertain in the creation theory because entrepreneurs must create opportunities (Foss et al., 2015). Therefore, it would not be a secure method to collect information, market changes, wealth creation, and technological advancement and analyze all these factors based on decision-making for opportunity creation. However, there must be the role of opportunity identification with the influence of entrepreneurial intention (Short et al., 2010; Curşeu and Schruijer, 2012; Kibler et al., 2014). Thus, we hypothesized the following hypotheses based on the discussion, and Figure 1 shows the study’s conceptual model.

H2a. There is a positive relationship between entrepreneurial intention and opportunity creation.

H2b. There is a positive relationship between GDMSs and opportunity creation.

H2c. The influence of entrepreneurial intention on opportunity creation will moderate by social capital such that upper levels of social capital will enhance the positive effect of entrepreneurial intention on opportunity creation.

## Materials and Methods

### Sample and Data Collection

This study used a quantitative research design, and the type of study was cross-sectional. To investigate the relationship between GDMS, entrepreneurial intention, opportunity discovery, opportunity creation, and social capital, we contacted the “Angel Investors,” which connects Pakistani entrepreneurs and investors. All the registered individuals of “Angel Investors” are the target population of this study. This forum helped us collect data from respondents, whereas the respondents of this study were investors looking to invest in an entrepreneurial opportunity. The probability systematic random sampling technique was used to collect data. The “Angel Investors” provided us with respondents’ e-mail contacts. We got approval and ensured that these data were only used for academic research purposes and kept confidential. Moreover, an e-mail was sent to entrepreneurs and investors attaching the softcopy of the survey form asking for their responses.

Furthermore, to minimize the chance of common method bias, we used a time-lagged approach for data collection (Podsakoff et al., 2012). The data collection practice consisted of two stages that took 80 days altogether; on the first stage, the data were gathered from 220 respondents, and on the second stage, the data were collected from 230 respondents. We sent a total of 600 e-mails to the entrepreneurs and investors on the addresses provided by the “Angel Investors,” on both stages, and we have received 450 responses with a response rate of 75%. After screening the collected information, we discarded 50 responses out of 450 and selected 400 answers for further analysis. As expected, all the investors were male by gender because we have a male-dominated society, and our women did not participate in entrepreneurial activities compared to the male gender.

The 40% of investors were aged between 18- and 50-year age group, and the remaining 60% were above the age of 50 years; it depicted that our young population is dependent on our elder people. The 50% of our respondents were investors with a qualification of matric and no University degree that was stunning for us, and only 15% were Master, M. Phil, or above. It means that the people investing and having entrepreneurial intention have no sound business study-related background. They may be belonged to a business family or have the agricultural income to invest in further entrepreneurial projects. Although educated people were very few in the list of investors, we selected as our study sample is based on probability. The 56% of investors that are more than half of the sample size have less than one year of investment experience, 20% have two years of experience, and only 9% of investors have investment experience up to five years, whereas 10% have experience of more than ten years and only there were 5% with the investment experience of more than ten years. From this information related to the investment experience, we have received two types of analysis. First, the more experienced investors have, they might be well settled and not looking for any more investment. The second is that the experienced investors were busier than inexperienced investors to answer our e-mail.

### Measures

This study has inducted the GDMSs (see Supplementary Appendix) as a composite variable consisting of different decision-making styles (rational, intuitive, dependent, avoidant, and spontaneous). These measurement constructs have 20 items, and this scale is adapted from the study of Krasniqi et al. (2019). This scale is already used and verified by Scott and Bruce (1995). A sample item, “I make decisions logically and systematically.” All the decision-making styles were measured using a 5-point Likert scale.

Moreover, the entrepreneurial intention was measured using five items on a 5-point Likert scale (ranging from 1 strongly disagree to 5 strongly agree). This measurement scale was adapted from the study of Liñán and Chen (2009). A sample item is that “I am ready to do anything to be an entrepreneur.”

Furthermore, we have adapted six items from the study (Weiss et al., 2019), and 5-point Likert scale was used. The measurement constructs, including structural social capital, relational social capital, and cognitive, social capital. This scale was also used by prior researchers (Andrews, 2010). A sample item “coordination and joint working with other departments is a major part of our approach to the organization of services.” Additionally, opportunity discovery was measured using five items adapted from Craig and Johnson (2006). This scale was tested and used by a previous study (Li et al., 2020b). Therefore, we have adapted this 5-point Likert scale. A sample item is that “I am excited by the knowledge that there are many unexploited entrepreneurial opportunities.” The questionnaire on opportunity creation contained six items and was based entirely on a 5-point Likert scale. This scale was adapted from Li et al. (2020b). A sample item is that “I am a source of innovative ideas.”

Demographic characteristics are critical to understanding data nature (Hsu et al., 2007). Therefore, we used age, gender, qualification, and investment experience to understand these characteristics’ expected association with entrepreneurial intention (Klyver and Schenkel, 2013).

## Results and Discussion

### Data Screening and Normality Test

We applied structural equation modeling (SEM) to analyze this research investigation’s multiple direct and indirect relationships. First, data assumptions have to be examined to determine the more effective SEM results because SPSS and AMOS software are susceptible to data normality. Therefore, we analyzed the data through multiple analytical tests such as cleaning, screening, normality, and outlier through the statistical tools package (Ainur et al., 2017). In addition, the Mahalanobis distance (D2) technique was also applied to check the outlier of the data (Jouan-Rimbaud and De Maesschalck, 2000). In the Mahalanobis test performed in AMOS, the values of (D2) were below the 59.3 thresholds for outlier; during the analysis of normality, our data achieved a given range of < 2 and < 7, respectively (Aggarwal, 2017). Therefore, the normality of data was valid for further analysis.

### Descriptive Statistics

Table 1 shows the sample data’s descriptive statistics, where the means, standard deviations (SDs), correlations, and scale reliabilities among all the variables were demonstrated. The correlation of an independent variable with a moderator and one dependent variable was significant. Therefore, the two approaches were applied to find out the multicollinearity evidence. First, we tested the Pearson’s correlation and standardized correlation through SEM, which was satisfactory. Second, the variance inflation factor (VIF) values are also presented in Table 1, which shows that there is no issue of multicollinearity in the data (Disatnik and Sivan, 2016).

**TABLE 1:** Means, SDs, correlations, and multicollinearity.

	Descriptive		Correlation					Collinearity statistics	
	Mean	*SD*	GDMS	SC	OD	EI	OC	Tolerance	VIF
Gender	1.00	0.00							
Age	3.35	0.91							
Qualification	2.00	1.22							
Investment experience	1.88	1.22							
Decision-making styles	3.77	0.88	**(0.98)**					0.857	1.167
Social capital	4.06	0.61	0.37**	**(0.82)**				0.861	1.161
Opportunity discovery	3.64	0.98	0.38**	0.17**	**(0.96)**				
Entrepreneurial intentions	3.60	0.86	0.07	−0.01	0.09	**(0.89)**		0.993	1.007
Opportunity creation	3.75	0.77	−0.04	0.04	0.03	0.12*	**(0.90)**		

n = 400, *p < 0.05; **p < 0.01.

*Scale reliabilities are presented within parentheses along the central diagonal.*

*Dependent variable: opportunity discovery, opportunity creation. The values with bold are the square root of the AVE.*

Furthermore, construct validity was assessed (Fornell and Larcker, 1981). Prior studies have remarked that this is widely used and more appropriate for finding discriminant validity (Alvarez and Barney, 2007; Li et al., 2020b). According to Fornell and Larcker (1981) criteria, the square root of the AVE is discriminant validity. Therefore, Table 1 findings show that all the values with diagonals were higher than the values of the correlation are discriminant validity, and below were correlations (Ab Hamid et al., 2017).

### Exploratory Factor Analysis

The step-by-step exploratory factor analysis (EFA) approach has been applied (Williams et al., 2010). Goldstein (1976) recommended the criteria for correlation matrix that could be produced through factor analysis; the most critical assumption is to measure the sample adequacy through the Kaiser–Meyer–Olkin (KMO) that was 0.73 and significantly higher the threshold of 0.70. Bartlett’s test of sphericity related to our study was significant, which provided the ground for factor analysis and supported the correlation matrix’s factorability. In the next step, we extracted the same five factors before rotation and used direct oblimin, the oblique rotation subdomain. As a result, we analyzed that the correlation among factors was not significant, then we reperform the factor rotation operation, but this time we selected orthogonal rotation with its subdomain of varimax (Hayton et al., 2004).

Therefore, Table 2 presents the results of factor rotation with each item communality extraction. All items are well extracted above the value of 0.40 with an average value of 0.70; those are significant. The loadings of items are above the value of 0.70. Furthermore, to evaluate the second assumption of construct validity, we calculated the convergent validity through the criteria of average variance extracted (AVE); all the values were good enough and reached above the threshold of 0.5 (Rosenbach et al., 2009). Thus, it is evident that all the construct’s reliability and validity achieved the acceptable threshold (Cronbach’s alpha > 0.70) composite reality > 0.80, and AVE > 0.50 (Bagozzi et al., 1991; Li et al., 2020a).

**TABLE 2:** Measurement model.

Construct	Items	Loadings	Communalities	α	CR	AVE
General decision-making styles	GDMS_1	0.781	0.620	0.910	0.980	0.666
	GDMS_2	0.822	0.732			
	GDMS_3	0.839	0.707			
	GDMS_4	0.795	0.679			
	GDMS_5	0.859	0.746			
	GDMS_6	0.766	0.636			
	GDMS_7	0.755	0.443			
	GDMS_8	0.801	0.680			
	GDMS_9	0.797	0.671			
	GDMS_10	0.752	0.449			
	GDMS_11	0.815	0.686			
	GDMS_12	0.826	0.716			
	GDMS_13	0.847	0.727			
	GDMS_14	0.814	0.698			
	GDMS_15	0.799	0.660			
	GDMS_16	0.841	0.731			
	GDMS_17	0.770	0.628			
	GDMS_18	0.838	0.718			
	GDMS_19	0.744	0.589			
	GDMS_20	0.786	0.602			
Opportunity discovery	OD_1	0.780	0.760	0.882	0.966	0.854
	OD_2	0.823	0.759			
	OD_3	0.797	0.784			
	OD_4	0.819	0.797			
	OD_5	0.866	0.843			
Opportunity creation	OC_1	0.867	0.783	0.845	0.903	0.608
	OC_2	0.831	0.754			
	OC_3	0.849	0.751			
	OC_4	0.878	0.817			
	OC_5	0.865	0.773			
	OC_6	0.881	0.656			
Entrepreneurial intentions	EI_1	0.878	0.820	0.834	0.900	0.646
	EI_2	0.876	0.781			
	EI_3	0.864	0.776			
	EI_4	0.826	0.735			
	EI_5	0.879	0.782			
Social capital	SC_1	0.843	0.778	0.763	0.836	0.564
	SC_2	0.828	0.720			
	SC_3	0.843	0.748			
	SC_4	0.822	0.700			
	SC_5	0.842	0.726			
	SC_6	0.792	0.705			

*Extraction method: principal component analysis. Rotation method: Varimax with Kaiser normalization.*

### Confirmatory Factor Analysis

Confirmatory factor analysis (CFA) was performed to check the model goodness-of-fit, and results were expressed in Table 3; the essential measure of model fit is the ratio of chi-square to degrees of freedom (CMIN/DF), and its value is 3.20, which does not fall under the interval of 1–3, but it is acceptable (Jiatong et al., 2021a). The root means a square error of approximation (RMSEA) is an absolute index calculated to evaluate the goodness-of-model fit. Its value is 0.09, which is not excellent but acceptable. The first relative measure of fit indices is the comparative fit index (CFI); its value is 0.91, which is not higher than 0.95, and it was terrible. Finally, the value of SRMR is also near the threshold but does not fall in the given criteria (Hu and Bentler, 1999). Thus, the overall model fit indices elaborate that this model is not reasonably fit, so the goodness-of-model fit is lacking, and it needs to be revised. As the modified measurement model to get the goodness-of-model fit, Table 3 elaborated on the modified indices, and all the measurements are significantly related to the given criteria.

**TABLE 3:** Measurement model fit indices.

Measure	Threshold	First model		Modified model	
		Estimate	Interpretation	Estimate	Interpretation
CMIN	–	3127.348	–	2385.968	–
DF	–	977	–	977	–
CMIN/DF	Between 1 and 3	3.200	Acceptable	2.442	Excellent
CFI	>0.95	0.910	Terrible	0.930	Acceptable
SRMR	<0.08	0.090	Acceptable	0.049	Excellent
RMSEA	<0.06	0.080	Terrible	0.060	Acceptable

### Common Method Bias

To find the issue of common method bias (CMB) in the data, we used the common latent factor (CLF) approach in AMOS (Chang et al., 2010). We calculated the standardized regression weights after adding the CLF, then removed CLF from the AMOS and calculated the standardized regression weights without CLF; the difference of the two measurements did not provide any value more than the (delta > 0.2) threshold. So, our data also achieved the second criterion and eliminated the influence of CMV bias before moving toward the structural model assessment (Williams and McGonagle, 2016).

### Assessment of Structural Model

Measuring the second-order moderated mediation through SEM in AMOS v.26 is a sophisticated method to approach the theoretical framework. This paper is based on model no 14, adopted from the templates of Hayes and Preacher (2013). However, the AMOS v.26 could not compute this model by default. Therefore, we developed the AMOS (machine language) syntax on equation no. 2 based on the template mentioned above to make it possible.

Syntax of model no.14 for two endogenous for user-defined estimates:

Conditional indirect effect of X on Y through

Mi = ai (b1i + b3iV)

Direct effect of X on Y = c’

1. First endogenous variable (opportunity discovery):
  IndEffLow1 = A*(B1 + (B3*0.75))
  IndEffMedium1 = A*(B1 + (B3*0))
  IndEffHigh1 = A*(B1 + (B3*0.75))
  Direct1 = C1
2. Second endogenous variable (opportunity creation):
  IndEffLow2 = A*(D1 + (D3*0.75))
  IndEffMedium2 = A*(D1 + (D3*0))
  IndEffHigh2 = A*(D1 + (D3*0.75))
  Direct2 = C2

**FIGURE 2:**
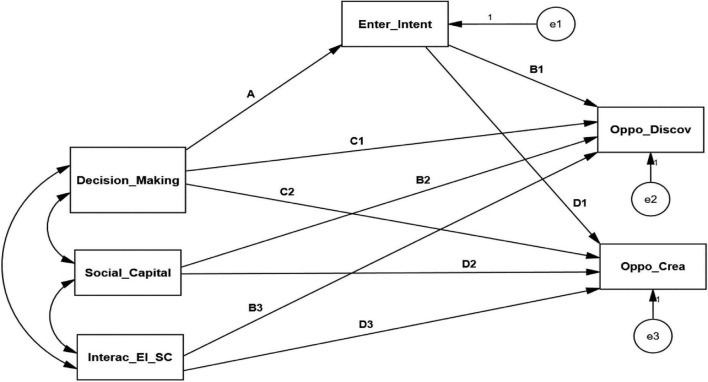
Structural model paths.

We have two endogenous variables based on this study literature, so we developed the two syntaxes and ran the second-order moderated mediation structural model with paths simultaneously, as shown in Figure 2. The syntax is supported to analyze the model simultaneously to measure the multiple direct and conditional indirect relationships, validated by some antecedent studies (González et al., 2017; Gill et al., 2021; Jiatong et al., 2021b). To solve the literature’s ambiguity, we give moderated mediation support to the theoretical framework; very considerable research has been developed (Hayes and Preacher, 2013).

### Testing of Hypothesis (Direct Effects)

**FIGURE 3:**
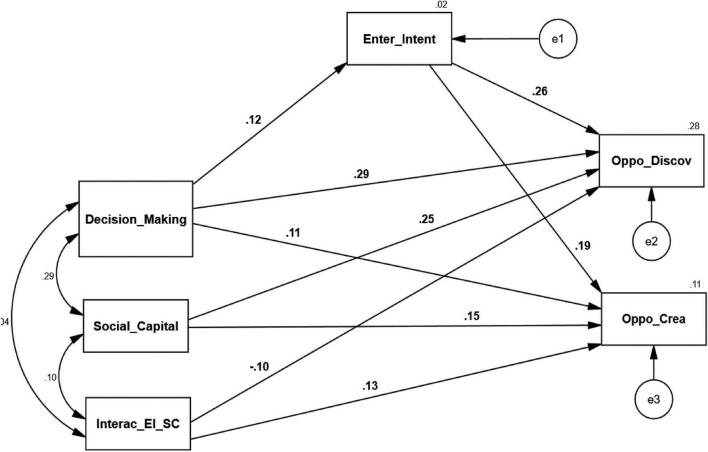
Structural model.

The direct effects with respective path labels are mentioned in Table 4 and Figure 3. Findings show that H1a GDMS has a positive and significant influence on entrepreneurial intention (β = 0.125, *p* = 0.005). Therefore, H1a was accepted. On path A, the decision-making styles directly impact entrepreneurial intention. The H1b also replicates the above scenario because the β = 0.262 and *p* = 0.000, which was significant. So, path B describes a significant direct relationship between entrepreneurial intentions and opportunity discovery. The H1c has a significant *p*-value and considerable (β = 0.285), so we accept this hypothesis.

**TABLE 4:** Hypothesis testing for the direct effects.

Direct effect		Structural model			Bootstrapping		Path
			
Hypothesis		*b*	*SE*	*p*	CI (LB) 95%	CI (UB) 95%	Label
H1a	Impact of GDMS on EI	0.125	0.046	0.005	0.238	0.160	A
H1b	Impact of EI on OD	0.262	0.058	0.000	0.120	0.389	B1
H1c	Impact of GDMS on OD	0.285	0.061	0.001	0.170	0.390	C1
H2a	Impact of EI on OC	0.186	0.062	0.001	0.060	0.308	D1
H2b	Impact of GDMS on OC	0.107	0.065	0.031	−0.009	0.222	C2

It means that path C1 indicates a positively significant direct relationship between the decision-making styles and opportunity discovery. The *p*-value of H2a is also significant, and the beta value is 0.186, so we accept this hypothesis. The D1 path demonstrates that entrepreneurial intentions positively directly relate to opportunity creation. Thus, H2 is a significant *p*-value of 0.31 and beta value of 0.107 that accept this hypothesis, where path C2 describes that the decision-making styles have a significant relationship with opportunity creation.

The direct relationship between the decision-making style and opportunity discovery is positively significant, whereas the direct relationship of decision-making styles with entrepreneurial intentions is also significant. The same situation exists between the decision-making styles and opportunity creation because their direct relationship is significant. So, the first two assumptions described by Hayes and Preacher (2013) related to the indirect effects of second-order moderated mediation have been achieved for both endogenous variables.

**FIGURE 4:**
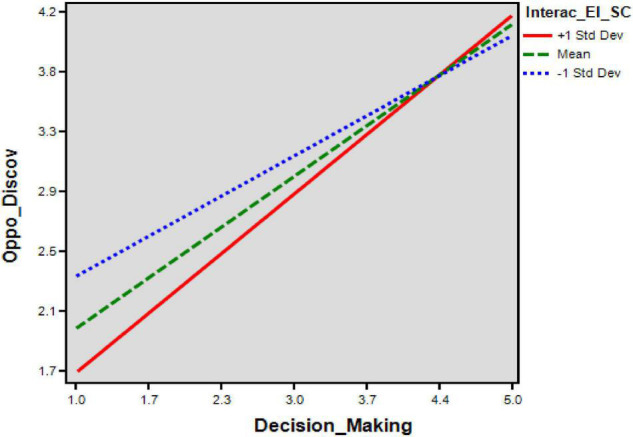
Three-way interaction on opportunity discovery.

The AMOS syntax was essential in evaluating this complex model with a single treatment through second-order CFA under the SEM approach (Muller et al., 2005). Table 5 shows the *p*-value of H1e (*p* 0.05 > 0.000; β = 0.262) is not accepted, with attaining the bootstrapping confidence interval also because zero does fall there. The interactional role of social capital with the entrepreneurial intentions on opportunity discovery is insignificant, presented as B3 path in Table 5 perhaps; the path D3 Figure 4 shows the significant indirect interactional effect of entrepreneurial intentions through social capital on opportunity creation. The social capital strengthens the positive relationship between entrepreneurial intentions and opportunity creation in Table 5; the second-order moderated mediation is positively significant. This study model has achieved the third assumption of Hayes and Preacher (2013), and he expressed that the indirect interactional effect of mediator and moderator has to be significant on endogenous but only for the opportunity creation. However, opportunity discovery has failed to achieve the third assumption (Bolin, 2014).

**TABLE 5:** Indirect and conditional effects (robust model).

Hypothesis	Bets (β)	*t*-value	*P*	Bias-corrected percentile 95% CI				Label
				Estimate	Lower	Upper	P	
H1d: EI × SC → OD	0.090	6.11	0.000	0.100	−0.224	0.021	0.056	B3
H2e: EI × SC → OC	0.241	3.89	0.000	0.133	0.013	0.268	0.011	D3

*SC, social capital; OD, opportunity discovery; OC, opportunity creation; GDMSs, general decision-making styles; EI, entrepreneurial intentions; β, standardized coefficient estimates; p, level of significance; Label, syntax; bootstrapping, 5,000; CI, confidence of interval 95%.*

### Testing the Conditional (Indirect Effect)

To analyze the fourth assumption of Hayes and Preacher (2013), this model has run through AMOS v.26 with 5,000 bootstrapping to get the assumption of user-defined estimates at a 95% level of confidence as it guided through syntax that this single robust model has to run for conditional indirect effect for two endogenous variables tested for social capital on high [−1 (0.75)], medium, and below [+1(0.75)]. Moreover, the results of Table 6 show that the opportunity discovery (β = 0.036, *p* < 0.000) indicates high levels of social capital (+1 SD) for investors: (β = 0.045, *p* < 0.000) indicate medium levels of social capital (0SD) for investors and (β = 0.053, *p* < 0.000) for low levels of social capital (−1 SD); thus, the fourth assumption has achieved for opportunity discovery. However, this study has rejected the H1e because it failed to achieve the third assumption. Therefore, the influence of entrepreneurial intentions on opportunity discovery is not moderated by social capital, such that upper levels of social capital levels do not enhance the positive effect of entrepreneurial intentions on opportunity discovery.

**TABLE 6:** Conditional indirect effects.

	β	Percentile 95% CI		*P*
		Lower bound	Upper bound	
**The conditional indirect effect at high, medium, and**				
**low social capital on opportunity discovery**				
Low (−1 SD) social capital	0.053	0.013	0.118	***
Medium (0) social capital	0.045	0.010	0.104	***
High (+ 1 SD) social capital	0.036	0.005	0.096	***
**The conditional indirect effect at high, medium, and**				
**low social capital on opportunity creation**				
Low (−1 SD) social capital	0.019	−0.004	0.064	***
Medium (0) social capital	0.030	0.004	0.083	***
High (+ 1 SD) social capital	0.041	0.006	0.104	***

Bootstrapping sample size = 5,000; β = standardized estimate. Significant ***p < 0.05.

**FIGURE 5:**
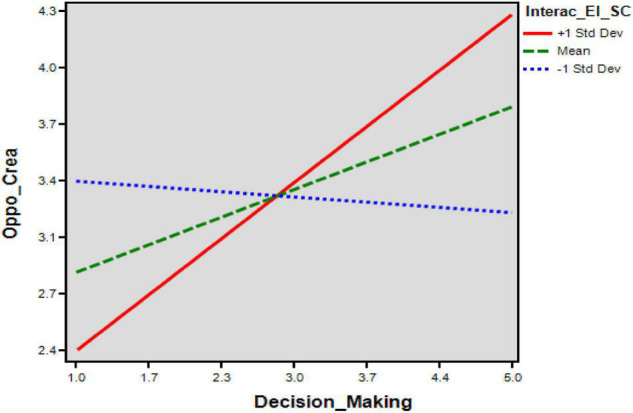
Three-way interaction on opportunity creation.

Finally, the conditional indirect effect of entrepreneurial intentions through social capital on opportunity creation resulted in variations. The results demonstrate in Figures 4, 5 that the degree of social capital for conditional indirect effect on opportunity creation varies for (+1 SD) high levels, (0SD) medium levels, and (−1 SD) low levels as (β = 0.041, *p* < 0.000), (β = 0.030, *p* < 0.000), and (β = 0.019, *p* < 0.000), respectively. Therefore, the influence of entrepreneurial intentions on opportunity creation moderates by social capital such that upper levels of social capital enhance the positive effect of entrepreneurial intentions on opportunity creation, strengthening investors’ level.

## Discussion

This study aimed to examine the role of GDMS on entrepreneurial intention, opportunity discovery, and opportunity creation through social capital. The direct hypotheses findings were statistically significant and align with the prior study of Alvarez and Barney (2007), who remarked that GDMS on entrepreneurial intention and opportunity discovery helps individuals to identify and exploit an opportunity because identification depends upon the prior knowledge of individuals, whereas exploitation depends upon the cognitive abilities of individuals, which leads to the discovery of new opportunities that form an entrepreneurial intention. Moreover, Krasniqi et al. (2019) suggests that GDMSs on entrepreneurial intention and opportunity creation usually are not assumed in the creation process, as they can be created by capabilities, actions, the enactment of entrepreneurs, and the exploration of ways to start a new business. Therefore, investors have more experience in handling business activities and performing day-to-day internal and external environment tasks to identify opportunity discovery and opportunity creation. Furthermore, these study results align with prior researchers who argue that entrepreneurial opportunities for the establishment of business ventures (Puhakka, 2007; Henley et al., 2017).

The relationship between discovery and creation is defined in prior literature (Sarasvathy et al., 2003; Alvarez and Barney, 2007). Moreover, previous researchers explain that discovery and creation lead to improved GDMSs and long-term influence on business performance (Foss and Klein, 2017). Therefore, discovery and creation are complementary theories, and both have to study together. Short et al. (2010) studied to distinguish the core features of opportunity discovery and creation theories to simply the knowledge constraints for effective business creation models, but they became unsuccessful, and the literature remained complex in this context. GDMSs became integral for individuals to start a business venture; indeed, our study also approved its significance related to business phenomena as many previous studies provided similar evidence (Krasniqi et al., 2019; Cristofaro et al., 2021; Sassetti et al., 2021). Our findings align with Henley et al. (2017), who argued that entrepreneurial intention is more important than entrepreneurial, analytical understanding that individuals can adopt through social interaction.

The GDMSs authenticate the managerial tendency and individual intentions to start a business venture to develop innovation through the lens of opportunity creation theory (Sassetti et al., 2021). The indirect interaction of social capital through entrepreneurial intentions significantly impacts GDMSs on opportunity creation and vice versa for opportunity discovery. That partially validates the findings of Weiss et al. (2019), who established the positive moderating role of social capital on entrepreneurial opportunities for business creation.

## Implications and Future Research Directions

The theoretical implications of our study suggest that it is not compulsory to study discovery with the creation of opportunity, but the theory of creation solely has the empirical influence for entrepreneurial opportunity creation. The theory of creation has an apparent and robust relationship with GDMSs where the entrepreneurial intentions have strengthened mediation role, and social capital enhances this relationship (González et al., 2017). This study extends the theoretical model of Krasniqi et al. (2019) GDMSs on entrepreneurial intention, opportunity discovery, and opportunity creation through the mediation of entrepreneurial intention and the moderation of social capital. This empirical examination contributes to entrepreneurship research literature and offers that the theory of discovery and creation could reduce the uncertainty and risk of the entrepreneurial general decision-making process.

This study provides practical implications for policymakers, investment organizations, and investors planning to invest in a new business venture in the healthcare sector. It helps them to understand the optimum use of their GDMSs compelling about opportunity discovery if they collect some information about the healthcare sector changes in KP and Punjab and entrepreneurial environment with their strong social ties. In this way, they can easily create opportunities rather than search for an unlimited opportunity discovery mission. The strong social interactions, especially at the regional level, would help them in opportunity creation, and the theory suggested that it is not imitative. Moreover, if they are not getting to connect with strong social ties, they can move to that region to have substantial social relations, which will help them to create entrepreneurial opportunities.

The government and public policymakers’ role is also crucial here; they could enhance entrepreneurial opportunities by providing a robust social environment for investors to encourage them to invest in the healthcare sector. Then, they become able to create entrepreneurial opportunities that will lift unemployment and create jobs. This study provides clear directions for investors to capture the healthcare sector as a future investment goal. The significant relation has explored healthcare entrepreneurial intentions, and this model establishes the fundamental framework for investors and managers.

As the regional social ties of KP and Punjab shall help investors invest in the healthcare sector, this opportunity has already been created by the government. The point is how entrepreneurs’ decision-making styles shall help them to implement opportunity creation theory by starting a business venture in healthcare. They can target underdeveloped areas such as small towns, tehsils, and communities by establishing the healthcare infrastructure. It would be a revolutionary decision of entrepreneurs; they would get profit on their investment, and on the other hand, they will produce tremendous employment opportunities that will boost the per capita income of local people and the country’s GDP.

This model provides a clear framework to investors that provide the confidence to start business ventures in the healthcare line, for example, small hospitals, medical laboratories, medical stores, and other associate segments. It will result in a well-integrated healthcare infrastructure that will reduce the mobility of the people from small towns and tehsils toward big cities. Consequently, it will reduce the pressure from urban healthcare infrastructure to enhance its capacity for the urban population. This study provides long-range planning for entrepreneurs based on the strategic vision of the federal government of Pakistan. This revolutionary planning of the healthcare sector has been appreciated by the World Health Organization (WHO) and exemplary legislation for developing countries to follow.

This study has some limitations. Our research focuses only on 400 healthcare investors of Pakistan. This study used self-report questionnaires that may lead to common method bias. Therefore, we suggest that future research conducts a longitudinal study on different samples with effectuation theory on opportunity discovery and opportunity creation to measure business performance or entrepreneurial action and contribute to the literature on entrepreneurship. This research also suggests that future researchers take entrepreneurial action as a dependent component to expand this study further. Simultaneously, GDMSs must be studied as independent again with its subdimensions in second-order to check the generalizability of our research. A broad avenue becomes open for entrepreneurial researchers and investors to enhance the body of knowledge on the multidimensional study of opportunity creation with entrepreneurial actions.

## Conclusion

This study aimed to provide an in-depth understanding of GDMSs on entrepreneurial intention and entrepreneurial theories such as discovery and creation. The relationships between these theories are less examined in the literature of entrepreneurship. This study provided evidence of which theory to study, the theory of discovery, and the theory of creation. At the same time, or these are mutually exclusive, this research has answered that these are not inverse but orthogonal theories and can be studied separately, and there is no empirical connection between these two theories. This research answered that social capital with strong ties enhances entrepreneurial opportunity exploitation. It simplifies the complex entrepreneurial opportunity creation model by bifurcating the association between the theories of discovery and creation. In regression analysis, there must be the probability of measurement error that makes the mediation analysis doubtful. Nevertheless, the step-by-step SEM implementation has removed this concern and made it a robust regression model. The study provides real-time implementable practical measures to strengthen the healthcare sector of Pakistan.

## Data Availability Statement

The raw data supporting the conclusions of this article will be made available by the authors, without undue reservation.

## Ethics Statement

The studies involving human participants were reviewed and approved by the Lyallpur Business School, Faisalabad, Pakistan. The patients/participants provided their written informed consent to participate in this study.

## Author Contributions

SG and MM proposed the research and wrote the manuscript. HJ and QA designed and carried out the experiments. JH and MS extensively edited and revised the manuscript. All authors contributed to the article and approved the submitted revised version.

## Conflict of Interest

JH was employed by company Shenzhen Shining Sea Asset Management Co., Ltd. The remaining authors declare that the research was conducted in the absence of any commercial or financial relationships that could be construed as a potential conflict of interest. The reviewer SA declared a shared affiliation with one of the authors, MM, to the handling editor at the time of review.

## Publisher’s Note

All claims expressed in this article are solely those of the authors and do not necessarily represent those of their affiliated organizations, or those of the publisher, the editors and the reviewers. Any product that may be evaluated in this article, or claim that may be made by its manufacturer, is not guaranteed or endorsed by the publisher.
