# Supplementary material for: Hunting the Best Opportunity Through the Arrow of General Decision-Making Styles: Unfolding the Role of Social Capital and Entrepreneurial Intention
Source: Front Psychol. 2022 Mar 3;13:814424. doi: 10.3389/fpsyg.2022.814424 (PMC8928583; doi:10.3389/fpsyg.2022.814424)
Supplement: Supplementary file 1 [file Data_Sheet_1.pdf]

# Appendix

| S.N | Code | Questions | SD | D | N | A | SA |
|---|---|---|---|---|---|---|---|
| **General Decision-Making Style GDMS** | | | | | | | |
| ***Rational*** | | | | | | | |
| 1 | GDMS1 | I double-check my information sources to be sure I have the right facts before making a decision. | 1 | 2 | 3 | 4 | 5 |
| 2 | GDMS2 | I make decisions in a logical and systematic way. | 1 | 2 | 3 | 4 | 5 |
| 3 | GDMS3 | My decision making requires careful thought. | 1 | 2 | 3 | 4 | 5 |
| 4 | GDMS4 | When making a decision, I consider various options in terms of a specific goal. | 1 | 2 | 3 | 4 | 5 |
| ***Intuitive*** | | | | | | | |
| 5 | GDMS5 | When making a decision, I rely upon my instincts. | 1 | 2 | 3 | 4 | 5 |
| 6 | GDMS6 | When I make decisions, I tend to rely on my intuition. | 1 | 2 | 3 | 4 | 5 |
| 7 | GDMS7 | I generally make decisions that feel right to me. | 1 | 2 | 3 | 4 | 5 |
| 8 | GDMS8 | When I make a decision, it is more important for me to feel the decision is right than to have a rational reason for it. | 1 | 2 | 3 | 4 | 5 |
| ***Dependent*** | | | | | | | |
| 9 | GDMS9 | I often need the assistance of other people when making important decisions. | 1 | 2 | 3 | 4 | 5 |
| 10 | GDMS10 | I rarely make important decisions without consulting other people. | 1 | 2 | 3 | 4 | 5 |
| 11 | GDMS11 | If I have the support of others, it is easier for me to make important decisions. | 1 | 2 | 3 | 4 | 5 |
| 12 | GDMS12 | I use the advice of other people in making my important decisions. | 1 | 2 | 3 | 4 | 5 |
| ***Avoidant*** | | | | | | | |
| 13 | GDMS13 | I avoid making important decisions until the pressure is on. | 1 | 2 | 3 | 4 | 5 |
| 14 | GDMS14 | I postpone decision making whenever possible. | 1 | 2 | 3 | 4 | 5 |
| 15 | GDMS15 | I often procrastinate when it comes to making important decisions. | 1 | 2 | 3 | 4 | 5 |
| 16 | GDMS16 | I generally make important decisions at the last minute. | 1 | 2 | 3 | 4 | 5 |
| ***Spontaneous*** | | | | | | | |
| 17 | GDMS17 | I generally make snap decisions. | 1 | 2 | 3 | 4 | 5 |
| 18 | GDMS18 | I often make decisions on the spur of the moment. | 1 | 2 | 3 | 4 | 5 |
| 19 | GDMS19 | I make quick decisions. | 1 | 2 | 3 | 4 | 5 |
| 20 | GDMS20 | I often make impulsive decisions. | 1 | 2 | 3 | 4 | 5 |
| **Entrepreneurial Intention** | | | | | | | |
| 21 | EI1 | Make every effort to start and run own firm. | 1 | 2 | 3 | 4 | 5 |
| 22 | EI2 | Professional goal is to become entrepreneur. | 1 | 2 | 3 | 4 | 5 |
| 23 | EI3 | Determined to create a firm. | 1 | 2 | 3 | 4 | 5 |
| 24 | EI4 | Ready to do anything to be entrepreneur. | 1 | 2 | 3 | 4 | 5 |
| 25 | EI5 | Very serious thought of starting a firm. | 1 | 2 | 3 | 4 | 5 |
| **Social Capital** | | | | | | | |
| 26 | SC1 | Staffs engage in open and honest communication with one another. | 1 | 2 | 3 | 4 | 5 |
| 27 | SC2 | Cross-departmental working is important in driving service improvement. | 1 | 2 | 3 | 4 | 5 |
| 28 | SC3 | Staffs keep each other informed at all times. | 1 | 2 | 3 | 4 | 5 |
| 29 | SC4 | There is a high level of trust between officers. | 1 | 2 | 3 | 4 | 5 |
| 30 | SC5 | Workers share the same ambitions and vision for the company. | 1 | 2 | 3 | 4 | 5 |
| 31 | SC6 | The authority's mission, values, and objectives are clearly and widely | 1 | 2 | 3 | 4 | 5 |

| | | | | | | | |
|---|---|---|---|---|---|---|---|
| | | understood and owned by all staff in the service. | | | | | |
| **Opportunity Discovery** | | | | | | | |
| 32 | OD1 | I discover entrepreneurial opportunities that exist in my industry. | 1 | 2 | 3 | 4 | 5 |
| 33 | OD2 | I discover previously unnoticed entrepreneurial opportunities. | 1 | 2 | 3 | 4 | 5 |
| 34 | OD3 | I am excited by the knowledge that there are many unexploited entrepreneurial opportunities. | 1 | 2 | 3 | 4 | 5 |
| 35 | OD4 | I undertake methods and solutions to product problems that build on my experience. | 1 | 2 | 3 | 4 | 5 |
| 36 | OD5 | I searched for product information and ideas that took the firm into existing product areas. | 1 | 2 | 3 | 4 | 5 |
| **Opportunity Creation** | | | | | | | |
| 37 | OC1 | I am attracted by the idea of breaking away from routine activity. | 1 | 2 | 3 | 4 | 5 |
| 38 | OC2 | I like to discover new ways of doing things. | 1 | 2 | 3 | 4 | 5 |
| 39 | OC3 | I prefer to find new uses for existing products. | 1 | 2 | 3 | 4 | 5 |
| 40 | OC4 | I am a source of innovative ideas. | 1 | 2 | 3 | 4 | 5 |
| 41 | OC5 | I collect information on totally new products. | 1 | 2 | 3 | 4 | 5 |
| 42 | OC6 | I search for product information involving experimentation and high risk. | 1 | 2 | 3 | 4 | 5 |
